# Neuronal clusterin expression is associated with cognitive protection in amyotrophic lateral sclerosis

**DOI:** 10.1111/nan.12575

**Published:** 2019-08-28

**Authors:** J. M. Gregory, E. Elliott, K. McDade, T. Bak, S. Pal, S. Chandran, S. Abrahams, C. Smith

**Affiliations:** ^1^ Centre for Clinical Brain Sciences University of Edinburgh Edinburgh UK; ^2^ Euan MacDonald Centre for Motor Neurone Disease Research University of Edinburgh Edinburgh UK; ^3^ Human Cognitive Neuroscience, Psychology University of Edinburgh Edinburgh UK

**Keywords:** ALS, cognition, ECAS, neuropathology, TDP‐43

## Abstract

**Aims:**

Clusterin is a topologically dynamic chaperone protein with the ability to participate in both intra‐ and extacellular proteostasis. Clusterin has been shown to be upregulated in the spinal cord of patients with amyotrophic lateral sclerosis (ALS) and has been shown to protect against TDP‐43 protein misfolding in animal and cell models. Previous studies have demonstrated an association between the pathological burden of TDP‐43 misfolding and cognitive deficits in ALS, demonstrating high specificity, but correspondingly low sensitivity owing to a subset of individuals with no evidence of cognitive deficits despite a high burden of TDP‐43 pathology, called mismatch cases.

**Methods:**

Hypothesizing that differences in the ability to cope with protein misfolding in these cases may be due to differences in expression of protective mechanisms such as clusterin expression, we assessed the spatial expression of clusterin and another chaperone protein, HspB8, in *post mortem* brain tissue of mismatch cases. We employed a modified in situ hybridization technique called BaseScope, with single cell, single transcript resolution.

**Results:**

Mismatch cases demonstrated differential spatial expression of clusterin, with a predominantly neuronal pattern, compared to cases with cognitive manifestations of their TDP‐43 pathology who demonstrated a predominantly glial distribution of expression.

**Conclusions:**

Our data suggest that, in individuals with TDP‐43 pathology, predominantly neuronal expression of clusterin in extra‐motor brain regions may indicate a cell protective mechanism delaying clinical manifestations such as cognitive dysfunction.

## Introduction

Amyotrophic lateral sclerosis (ALS) is a progressive neurodegenerative disease characterized by muscle denervation and atrophy leading to paralysis and death. These motor features are often accompanied by cognitive dysfunction in as many as 50% of ALS patients with up to 15% of patients exhibiting features of frontotemporal dementia (FTD) 1, 2, 3. Despite this clinical heterogeneity, a unifying pathological feature seen in the majority of ALS and FTD patients is the presence of the 43 kDa Tar‐DNA binding protein (TDP‐43), whose pathological misfolding and accumulation is observed in the brains and spinal cord of the majority of ALS patients with the notable exception of ALS cases caused by *SOD1* mutation 4. TDP‐43 is a predominantly nuclear protein involved in transcriptional regulation. In ALS *post mortem* brain and spinal cord tissue, TDP‐43 is found to be pathologically phosphorylated and C‐terminally truncated in cytoplasmic aggregates 5. The toxicity conferred by TDP‐43 in ALS is thought to be partly loss‐of‐function caused by sequestration in insoluble aggregates limiting transcriptional regulation, and partly due to a gain‐of‐function where cytotoxic cytoplasmic aggregates can lead to the dysregulation of proteostasis and sequester other aggregation prone proteins causing further cytotoxicity and contributing to cell death 6.

Protein misfolding, aggregation and subsequent cell death are common pathways in all neurodegenerative diseases exhibiting cognitive phenotypes. However, cognitive defects in ALS, while common, typically affect multiple domains and there is a distinctive cognitive profile in ALS compared to other neurodegenerative diseases, and cognition is therefore best assessed by an ALS specific cognitive test such as the Edinburgh Cognitive ALS Screen (ECAS). The ECAS is a neuropsychological assessment specifically designed to assess the cognitive deficits associated with ALS, notably (i) verbal fluency, (ii) executive function and (iii) language 7. It also comprises assessment of visuospatial and memory assessments that are typically disordered in other neurodegenerative diseases such as Alzheimer's disease. The ECAS provides high sensitivity and specificity using published cut off scores for abnormality (7). ECAS has been validated extensively in many different diverse populations of ALS and FTD patients 8, 9, 10, 11 and against other neuropsychological batteries 12, 13.

While several studies have shown an association between TDP‐43 pathology and cognitive deficits, these studies have consistently identified a subgroup of patients who have abundant TDP‐43 pathology and no corresponding cognitive defects 14, 15. These cases are called mismatch cases, due to the mismatch between the abundant pathology and corresponding lack of clinical manifestations. Importantly, previous studies have overlooked protective mechanisms that may be at play in these cases. In response to protein aggregation and subsequent dysregulation of proteostasis, cellular mechanisms, including the upregulation of chaperone proteins, are initiated to reduce the cytotoxic burden of these misfolded proteins. Our hypothesis, therefore, is that the heterogeneity seen in the cognitive and pathological manifestations of ALS, may, in part reflect individual differences in the balance between proteotoxic stress and protective mechanisms. The aim of this study was to characterize the expression of two such putative protective mechanisms, the expression of two chaperone proteins, clusterin and HspB8, known to protect against TDP‐43 pathology 16, 17. Clusterin responds to protein misfolding stress by retrotranslocating from its normal extracellular distribution to the intracellular compartment 18, where it can directly interact with TDP‐43, (i) preventing its aggregation, and (ii) restoring TDP‐43 to its normal nuclear localization 16. HspB8 is an intercellular chaperone protein, whose role in proteostasis is well‐established in reducing TDP‐43‐associated proteotoxicity 16, 19, 20.

Within our cohort of ALS brain bank patients, 27 patients had undergone standardized cognitive testing using ECAS during life. Of these 27 patients, following in depth neuropathological assessment of TDP‐43 pathology, we were able to identify six patients who fit the classification of a mismatch case, with abundant TDP‐43 pathology and a normal ECAS score. For comparison, we identified 11 cases who had TDP‐43 pathology with evidence of mild cognitive deficits. Within this cohort we analysed the spatial expression of clusterin and HspB8, with single cell, single transcript resolution, employing a novel modified *in situ* hybridization technique called BaseScope. In doing this, we demonstrate spatial expression differences in clusterin between cases with cognitive deficits and mismatch cases without cognitive manifestations, highlighting an important protective mechanism.

## Methods

### Case identification

We identified, from the Medical Research Council (MRC) Edinburgh Brain Bank, a cohort of 17 ALS patients for neuropathological assessment, all of whom had undergone standardized cognitive testing using the ECAS and demonstrated TDP‐43 in nonmotor brain regions at *post mortem*. In our analysis, we sought to assess protective mechanisms (cell‐type specific clusterin and HspB8 expression). All clinical data including the ECAS were collected as part of Scottish Motor Neurone Disease Register (SMNDR) and Care Audit Research and Evaluation for Motor Neurone Disease (CARE‐MND) platform (ethics approval from Scotland A Research Ethics Committee 10/MRE00/78 and 15/SS/0216) and all patients consented to the use of their data during life. Control brains were selected from the sudden death brain bank and therefore have not died of a chronic illness or neurological condition. All control cases are rigorously assessed by experts, both clinically and neuropathologically, to rule out any evidence of disease, including the use of an extensive standardized panel of immunohistochemistry, with pathology being assessed using well‐defined and published grading systems. All *post mortem* tissue was collected via the Edinburgh Brain Bank (ethics approval from East of Scotland Research Ethics Service, 16/ES/0084) in line with the Human Tissue (Scotland) Act. Use of human tissue for *post mortem* studies has been reviewed and approved by the Edinburgh Brain Bank ethics committee and the Academic and Clinical Central Office for Research and Development (ACCORD) medical research ethics committee (AMREC).

### TDP‐43 neuropathological analysis

Brain tissue was taken at *post mortem* from standardized Brodmann areas (BA) relating to cognitive dysfunction in ALS (BA44/45 was selected for its role in language and fluency function and BA46 for its role in executive and fluency) and fixed in 10% formalin for a minimum of 24 h. Tissue was dehydrated in an ascending alcohol series (70–100%) followed by three successive 4‐h washes in xylene. Three successive five‐hour paraffin wax embedding stages were performed followed by cooling and sectioning of the formalin‐fixed paraffin embedded (FFPE) tissue on a Leica microtome in 4‐μm sections on to a superfrost microscope slide. Sections were dried overnight at 40°C and immunostaining was performed using the Novolink Polymer detection system with the proteintech anti‐phospho(409‐410)‐TDP‐43 antibody at a 1 in 1000 dilution and DAB chromogen and counterstained with haematoxylin, according to standard operating procedures. As previous studies have demonstrated no difference in or effect of relative abundance of TDP‐43 pathology with cognitive manifestations 11, the slides were reviewed by two independent pathologists for presence or absence of TDP‐43 pathology only. Cell morphology was used to differentiate (i) glial cells (with small 4–5 µm) oval to round nuclei and a small quantity of surrounding cytoplasm. Neurons were classified as such if they had larger nucleus (5–10 µm) and a larger quantity of blue cytoplasm forming peripheral processes (stained using haematoxylin counterstain).

### BaseScope analysis – assessing spatial expression of clusterin and HspB8

FFPE tissue was sectioned at 4 μm thickness on to superfrost slides. BaseScope reagents (*Advanced Cell Diagnostics*) were used as per the manufacturer's guidelines according to the original protocol 21. In brief, following deparaffinization, tissue sections were incubated with hydrogen peroxide for 10 min at room temperature and target antigen retrieval was performed by submerging slides in BaseScope 1X target retrieval reagent at 99°C in a Braun Multiquick FS 20 steamer for 15 min. The tissue was then permeabilized using BaseScope protease III at 40°C for 30 min. Probe hybridization was then performed by incubating the slides with four drops of custom designed BaseScope probe (to recognize clusterin/HspB8 mRNA transcripts), negative control probe (DapB) or positive control (PPIB) probe for 2 h at 40°C. Following successive probe amplification steps, transcripts were detected using the BaseScope RED detection kit and slides were counterstained using haematoxylin and lithium carbonate. The slides were then cleared in xylene and mounted with a 24 × 50 mm coverslip using two drops of VectaMount mounting medium. Sections were then imaged at 20x magnification on a NanoZoomer slide scanner and relative abundance of transcripts was quantified by two independent pathologists. One‐way ANOVA (with Bonferroni correction for multiple comparisons) was used to analyse clusterin expression (Figure 2).

## Results

### Subgroups of patients exhibiting TDP‐43 pathology with no cognitive impairment have differential spatial expression of clusterin

Within our cohort of 27 patients who had undergone ECAS testing during life, we identified a subgroup of six patients that demonstrated no cognitive impairment when assessed by the ECAS, but did exhibit extra‐motor TDP‐43 pathology when assessed at *post mortem*, so called mismatch cases, and 11 patients who had TDP‐43 and corresponding cognitive dysfunction; one with executive dysfunction, two with fluency dysfunction, six with language dysfunction and two with a mixed cognitive deficit (Table 1). The median survival (from onset of disease) of the mismatch cases, 71 months (range: 16–159), was not statistically significantly different from individuals where the ECAS did accurately predict TDP‐43 pathology, 58 months (range: 13–150) (Mann–Whitney *U* test: *U*‐value = 26; *Z*‐score = −0.65327; *P* = 0.5157), suggesting that disease duration was not likely to be playing a significant role in the clinical manifestations of TDP‐43 pathology in these cases. Furthermore, the time between ECAS testing and death was not significantly different between the two groups. The group with no cognitive impairment was comprised of four individuals assessed within one year of death and two individuals with more than a one‐year gap between ECAS testing and death. The group with cognitive impairment was comprised of six individuals assessed within one year of death and five individuals with more than a one‐year gap between ECAS testing and death. Fisher's exact test revealed no significant difference between these two groups as the two‐tailed p value equates to 0.6004. Two mismatch cases had an underlying genetic mutation, both of which were a *C9orf72* hexanucleotide repeat expansion (30%), compared to five genetic mutations (four *C9orf72* and one *NEK1* mutation) in the 11 comparison cases with cognitive deficits (45%). Fisher's exact test revealed no significant difference between these two groups as the two‐tailed p value equates to 1.0000.

**Table 1:** Summary of cohort demographics including genetics, median survival from symptom onset, time from ECAS to PM and gender

	TDP‐43 pathology with no evidence of cognitive dysfunction (mismatch cases)	TDP‐43 pathology with evidence cognitive dysfunction	Statistics
	(*n* = 6)	(*n* = 11)	
Genetics	*C9orf72*: 2 cases	*C9orf72*: 4 cases	Fisher's exact test *P* = 1.0000
		*NEK1*: 1 case	
Median survival (from symptom onset)	71 months (range: 16–159)	58 months (range: 13–150)	Mann–Whitney *U*: *P* = 0.5157
Time from ECAS to PM	Four individuals assessed within one year of death and two individuals with more than a one year gap between ECAS testing and death.	Six individuals assessed within one year of death and five individuals with more than a one year gap between ECAS testing and death.	Fisher's exact test *P* = 0.6004.
Gender	Male = 4	Male = 7	Fisher's exact test *P* = 1.0000
	Female = 2	Female = 4	

Given that these mismatch cases had pathologically misfolded TDP‐43 in extra‐motor brain regions without suffering clinical manifestations at the time of ECAS assessment during life, we wanted to investigate if they had upregulation of compensatory, protective mechanisms that could account for this altered protein misfolding capacity and which would allow for this high burden of protein misfolding to be tolerated. We assessed the expression of two chaperone proteins: clusterin and HspB8, both known to protect neurones from intracellular protein misfolding stresses, and more specifically known to protect against TDP‐43 proteotoxicity 16, 17. For this analysis, we selected the same brain regions as above; BA46 and BA44/45.

**Figure 1:**
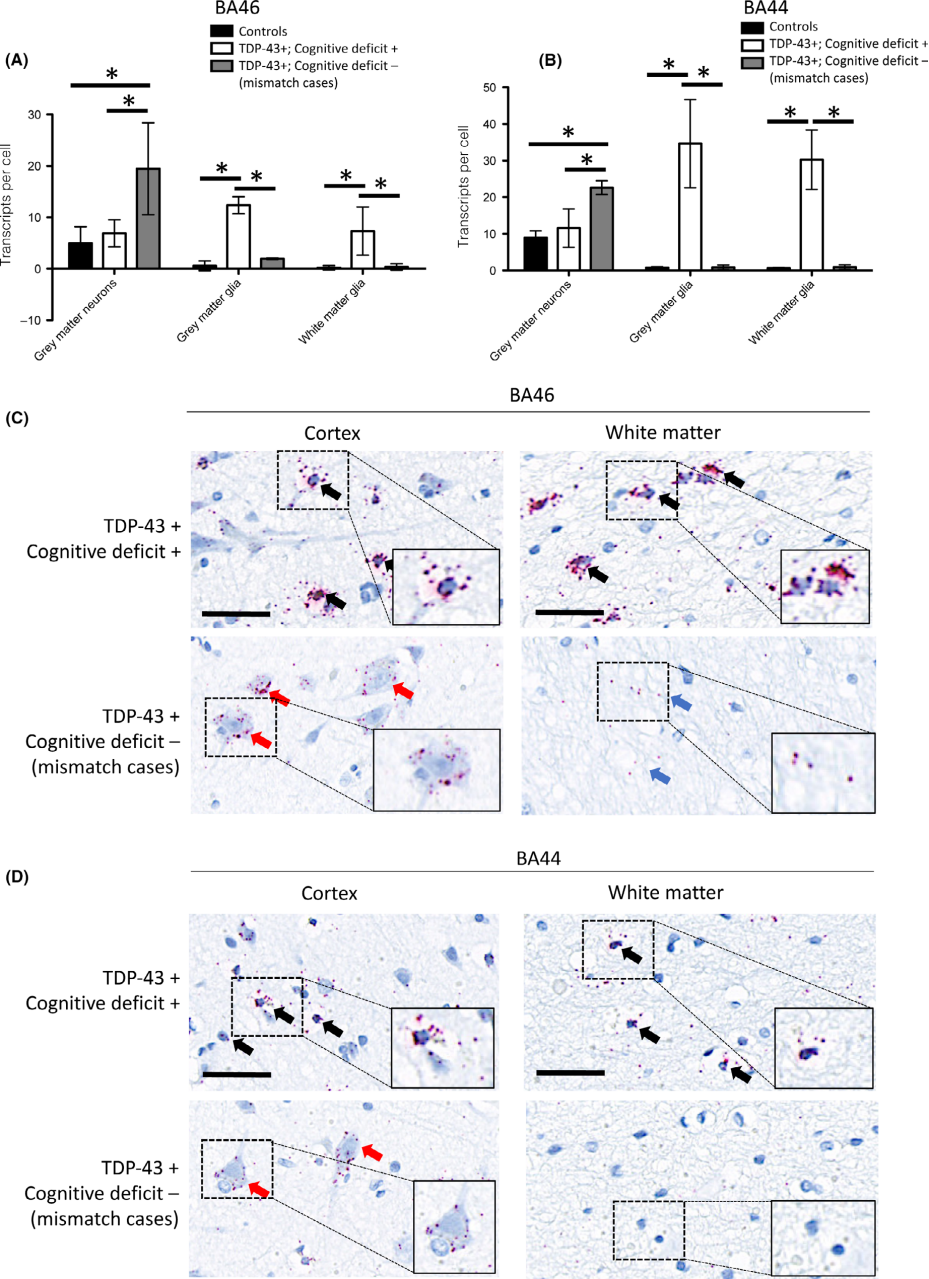
Individuals with TDP Pathology but no associated cognitive impairment exhibit predominantly neuronal expression of clusterin. (**A**). Graph quantifying grey matter neuronal and glial expression and white matter glial expression of clusterin, in the BA46 region associated with executive function, in cases with TDP‐43 pathology and cognitive deficits (TDP‐43+, Cognitive deficit+), mismatch cases (TDP‐43+, Cognitive deficit‐) and three non‐ALS/non‐neurological control cases (TDP‐43‐, Cognitive deficit‐); demonstrating upregulation of clusterin in all ALS cases, with predominantly glial expression in cases with TDP‐43 pathology and corresponding cognitive deficits and predominantly neuronal expression in mismatch cases. *Indicates *P* < 0.001, one‐way ANOVA with Bonferroni correction for multiple comparisons (*n* = 3 patients per group; 10 cells in each of 3 randomly selected high power fields of view were assessed per case). (**B**) Same as A but assessing the BA44 region associated with language and fluency. *Indicates *P* < 0.001, one‐way ANOVA with Bonferroni correction for multiple comparisons (*n* = 3 patients per group; 10 cells in each of 3 randomly selected high power fields of view were assessed per case). (**C**) Representative region of the brain associated with executive function (BA46), demonstrating striking difference in expression of the chaperone protein clusterin between cases with and without cognitive dysfunction. Black arrows indicate glial expression, red arrows indicate neuronal expression, blue arrows indicate neuropil staining that is not associated with glial cells consistent with neuronal expression. Scale bars = 50 µm. (**D**) Representative region of the brain associated with language and fluency (BA44), demonstrating striking difference in expression of the chaperone protein clusterin between cases with and without cognitive dysfunction. Black arrows indicate glial expression, red arrows indicate neuronal expression and blue arrows indicate neuropil staining that is not associated with glial cells consistent with neuronal expression. Scale bars = 50 µm.

**Figure 2:**
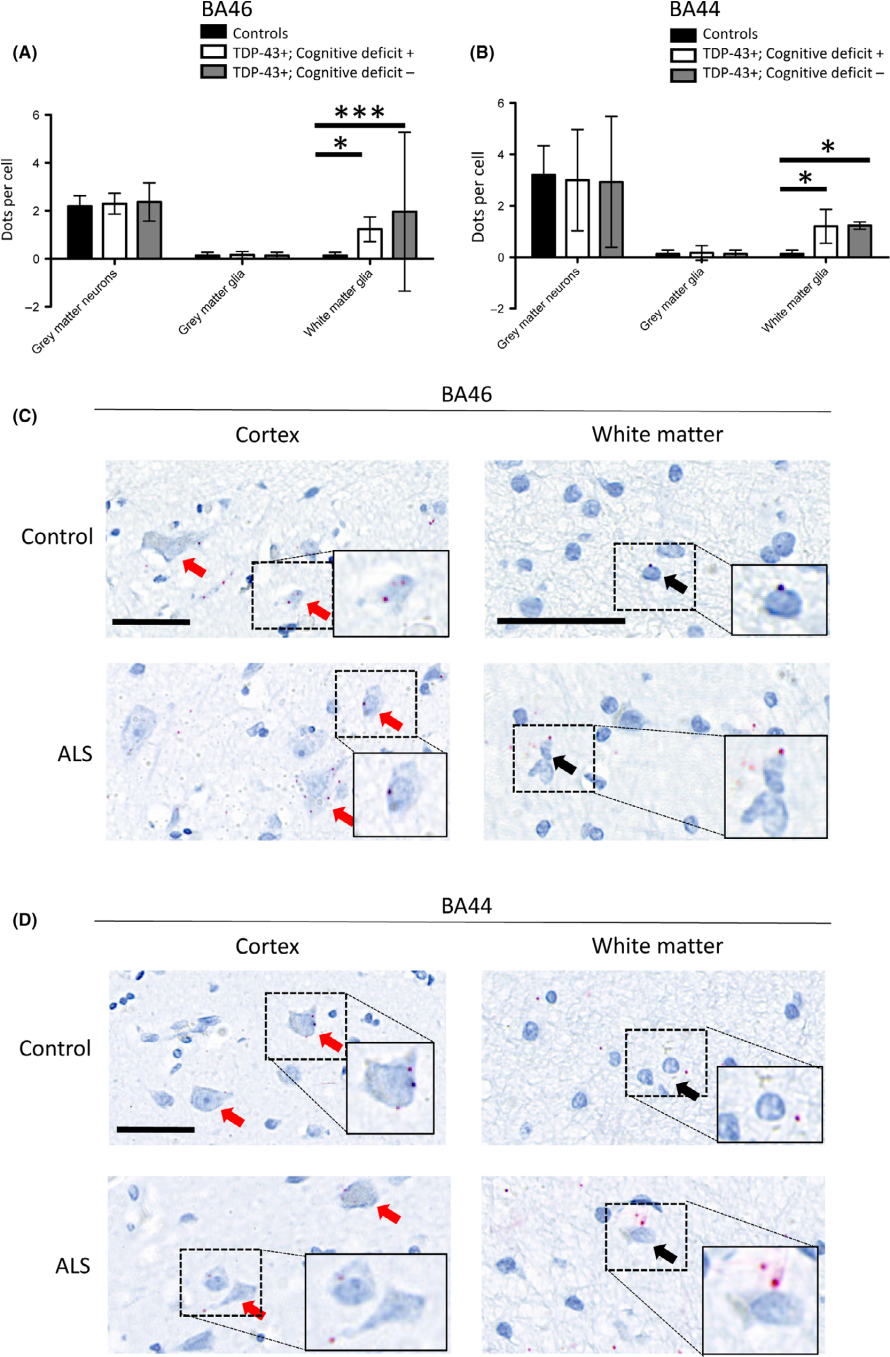
HspB8 expression is upregulated in the white matter glia of ALS patients compared to controls. (**A**) Graph quantifying grey matter neuronal and glial expression and white matter glial expression of HspB8, in the BA46 region associated with executive function, in cases with TDP‐43 pathology and cognitive deficits (TDP‐43+, Cognitive deficit+), mismatch cases (TDP‐43+, Cognitive deficit‐) and three non‐ALS/non‐neurological control cases (TDP‐43‐, Cognitive deficit‐); demonstrating upregulation of HspB8 in the white matter glia of all ALS cases exhibiting TDP‐43 pathology. ***Indicates *P* < 0.001, *Indicates *P* < 0.05, one‐way ANOVA with Bonferroni correction for multiple comparisons (*n* = 3 patients per group; 10 cells in each of 3 randomly selected high power fields of view were assessed per case). (**B**) Same as A but assessing the BA44 region associated with language and fluency. *Indicates *P* < 0.05, two‐way ANOVA with Bonferroni correction for multiple comparisons (*n* = 3 patients per group; 10 cells in each of 3 randomly selected high power fields of view were assessed per case). (**C**) Representative region of the brain associated with executive function (BA46), demonstrating neuronal (red arrows) and glial (black arrows) expression of HspB8, with increased HspB8 expression within white matter glia. Scale bars = 50 µm. (**D**) Representative region of the brain associated with language and fluency (BA44), demonstrating neuronal (red arrows) and glial (black arrows) expression of HspB8, with increased HspB8 expression within white matter glia. Scale bars = 50 µm.

As previously demonstrated, clusterin expression was increased in all ALS cases assessed compared to age‐ and sex‐matched controls with no clinical or pathological evidence of neurological disease (Figure 1 **A**,**B**) 16. In the mismatch cases, there is high expression of clusterin in the neuronal cells, with correspondingly low levels of expression in the glial cells (Figure 1). This is strikingly different to the individuals with TDP‐43 pathology who do have cognitive deficits, as these individuals have high levels of glial expression, with very little neuronal expression (Figure 1 **C**,**D**). Cell‐type specific expression of clusterin was quantified in representative brain regions using BaseScope analysis, allowing for the direct visualization of single mRNA transcripts at a single cell level. This revealed a statistically significant increase in expression of neuronal clusterin in mismatch cases (*P* < 0.001, two‐way ANOVA with Bonferroni correction for multiple comparisons) and a statistically significant increase in expression of glial clusterin in cases with TDP‐43 pathology with a cognitive deficit (*P* < 0.001, two‐way ANOVA with Bonferroni correction for multiple comparisons; Figure 2 **A,B**). HspB8 is another chaperone protein, which was used as a comparator whose intracellular role in proteostasis is well‐established in reducing TDP‐43‐associated proteotoxicity 17, 19, 20. However, HspB8, while statistically significantly upregulated in the white matter glia of ALS cases with TDP‐43 pathology compared to non‐neurological controls (Figure 2 **A**,**B**), unlike clusterin, was not differentially regulated between individuals with or without cognitive deficits. Furthermore, given the differential expression pattern of HspB8 was noted to be in the white matter glia, this pattern more likely reflects reactive gliosis and unlike clusterin is not spatially dysregulated between cognitively impaired and cognitively intact individuals (Figure 2 **C**).

## Discussion

While there is evidence to suggest a statistically significant association of cognitive deficits in ALS with TDP‐43 pathology 14, 15, in these published datasets there is a subset of individuals with evidence of substantial TDP‐43 pathology with no evidence of cognitive dysfunction, so called mismatch cases. Indeed, these mismatch cases are not unique to the clinical phenotype caused by TDP‐43 pathology as similar mismatch cases have been identified in other cohorts including Alzheimer's cases with substantial misfolded amyloid and tau deposits, with no concomitant cognitive deficits and the ALS‐PDC cluster on Guam where there were cases with substantial tau neurofibrillary tangles with no associated cognitive deficits 22, 23. Our findings advance the field further by demonstrating the importance of looking not just at the pathological processes (TDP‐43 misfolding) but also assessing the opposing, potentially protective mechanisms (e.g. clusterin expression) that some individuals mount to enable them to cope with the apparent neurotoxic insult of protein misfolding. The ability to express high levels of neuronal clusterin, could be affording these cells some neuroprotection and reducing the clinical manifestations of the disease that would be associated with neurotoxicity of TDP‐43 accumulation in these regions. High glial expression of clusterin in the patients with TDP‐43 pathology and cognitive deficits could also reflect high levels of reactive gliosis resulting from axonal degeneration and cell death. Moreover, taking account of the HspB8 results, these findings suggest that the cell‐type specific dysregulation of clusterin distinguishes between individuals with or without cognitive deficits and is specific to clusterin and not simply reflecting an underlying reactive process. However, given this is *post mortem* tissue, it is uncertain whether the differential expression of clusterin is (i) the cause of, or (ii) a consequence of the different cognitive‐pathological phenotypes, although, it is clear, that there are striking differences in putative protective cellular mechanisms in these patients.

Stratifying patients for clinical trials based on cognition, would be beneficial as the high specificity would result in only patients with definite TDP‐43 pathology being recruited. However, this approach, owing to reduced sensitivity, would fail to identify mismatch cases, that could also benefit from therapies targeting TDP‐43 misfolding. Our findings here, suggest that these mismatch cases could instead be identified by clusterin phenotyping, in addition to cognitive testing, as, the striking differential expression of clusterin could be a target for biomarker development. Clusterin is a normally secreted chaperone protein, with the ability to retrotranslocate to the cytosol under conditions of intracellular stress, such as TDP‐43 misfolding and accumulation 16. In fact, clusterin levels in both cerebrospinal fluid (CSF) and plasma have been investigated for other neurodegenerative diseases and a recent mass spectrometry analysis of plasma from patients with ALS showed that clusterin levels were able to distinguish between non‐neurological controls and cognitively impaired ALS patients 24, 25. It is therefore possible that clusterin could be used in conjunction with cognitive testing with tests such as the ECAS, to improve the sensitivity of the detection of TDP‐43 pathology in nonmotor brain regions, in ALS patients and improve (i) prognostication regarding risk of developing cognitive impairment and (ii) stratification for clinical trials.

## Author contributions

LE, JG, CS and SA contributed to (i) conception and design of the study, (ii) acquisition and analysis of data and (iii) drafting a significant portion of the manuscript or figures. KM contributed to (i) conception and design of the study, (ii) acquisition and analysis of data. TB, SC and SP contributed to (i) conception and design of the study and (ii) drafting a significant portion of the manuscript and figures.

## Disclosure

The authors declare no conflicts of interest.
